# Simulation and Optimization Strategy of Storm Flood Safety Pattern Based on SCS-CN Model

**DOI:** 10.3390/ijerph19020698

**Published:** 2022-01-08

**Authors:** Xinhong Cai, Dawei Xu

**Affiliations:** 1 College of Landscape Architecture, Northeast Forestry University, Harbin 150000, China; xinhongcai96@gmail.com; 2 Key Lab for Garden Plant Germplasm Development & Landscape Eco-Restoration in Cold Regions of Heilongjiang Province, Harbin 150000, China

**Keywords:** rain and flood safety pattern, SCS model, hydrological spatial analysis, urban disaster prevention planning, Harbin central district

## Abstract

The contradiction between rapid urbanization’s demand for land resources and the ecological environment is increasing, which has led to large-scale hardening of the underlying surface of the city and reduction of land for storage. In addition, construction land occupies rainwater confluence land, resulting in a significant decline in urban stormwater control capabilities. The increasingly frequent flood disasters in recent years have exposed the contradiction between urban construction and stormwater safety that cannot be ignored. Therefore, this article takes the central city of Harbin as the research object, uses ArcGIS for spatial analysis and SCS (Soil Conservation Service) hydrological model simulation to construct the rain and flood safety pattern in the research area, and proposes targeted optimization suggestions and strategies based on the evaluation results to achieve the purpose of coordinating the water ecosystem service function with social and economic development. The research shows that protecting the original stormwater corridor and strengthening the connection between the stormwater control patches can effectively guarantee the connectivity of the stormwater corridor, build the natural stormwater regulation and storage system, and then increase the ability of the city to resist the risk of rainstorm, reduce the disaster caused by urban waterlogging, and achieve the goal of sponge city construction.

## 1. Introduction

As an important strategic resource of regional development, water is closely related to the social economy and human settlements; it is a key element of sustainable urban development. Building a secure and harmonious territorial space pattern is a long-term strategic goal set by 2035 which was determined in the “Several Opinions of the Central Committee of the Communist Party of China and the State Council on Establishing a Land and Space Planning System and Supervising Implementation” [1]. The new pattern of territorial space development and protection is proposed at a historical moment when China is about to complete the building of a moderately prosperous society and embark on a new journey of building a modern socialist country in all respects. The strategic goals of coordinated regional development and new-type urbanization are of great significance. However, in 2021, the occurrence of the devastating floods in Zhengzhou and the occurrence of heavy rain disasters in Shanxi Province forced the implementation of the goal of building a security pattern to accelerate. Among the various disasters and accidents that threaten the security of national land and space, floods are one of the disasters with the highest frequency, the widest area affected, and the most sensitive response to urban planning and construction [2].

With the continuous deterioration of global climate problems in recent years, under the interaction of climate change, population growth, and rapid urbanization, the vulnerability of urban systems in the face of natural disasters has been revealed [3]. Under the influence of this, rain and flood disasters caused by extreme rainfall have occurred frequently, causing huge property losses. According to statistics from the UN Office for Disaster Reduction, one-third of the world’s natural disasters and economic losses are related to floods [4]. According to incomplete statistics, the world’s annual property losses caused by rain and flood disasters account for about 40% of natural disasters. In China, there are more than 100 million people affected annually. The economic losses caused by urban and rural flood disasters can reach up to the GDP 1–2% [5]. From the perspective of the causes of flood disasters, in addition to the increase in the probability and frequency of extreme rainfall events caused by climate change, along with the acceleration of urbanization, a large area of hardened construction land has increased, and the underlying surface of the city has hardened on a large scale. The reduction of storage land weakens the function of natural underlays to store rainfall in the face of rainfall, resulting in a significant decline in urban rain and flood control capabilities and increasing peak water volume and waterlogging risks [6]. In addition, the guiding ideology of China’s existing urban drainage system is basically to “quick dry and fast drainage” along the roads and pipe networks, leading to a significant increase in the risk of waterlogging in the lower reaches of the city and in low-lying areas [7]. Moreover, spatial planning is an important way to affect the distribution of disaster-bearing people, the layout of buildings and lifelines, and the pattern of agricultural production [8]. Reasonable planning and construction affects the occurrence of flood disasters on the one hand and is also restricted by flood disasters on the other hand.

A reasonable evaluation of the city’s rain and flood safety pattern is not only a theoretical requirement for assessing the city’s ability to resist and recover from rain and flood disasters but also a realistic demand for promoting the implementation of a “sustainable development” policy. Our work in this article is as follows: (1) Firstly, we discussed the importance of the construction of rain and flood safety pattern on the impact of urban safety. Besides, taking the waterfront city of Harbin as an example, the SCS (Soil Conservation Service) model is used to simulate the runoff and carry out quantitative research to simulate the rain and flood safety pattern in the central city of Harbin and propose corresponding improvement strategies based on the simulation results, which will help with targeted measures to carry out sponge city construction; (2) Harbin is a cold city located in northeastern China. The simulation study of rain and flood safety patterns for winter cities can effectively fill the gaps in current research on winter cities; (3) The simulation results were evaluated, and based on these results, suggestions were made to improve the optimization of the urban rain and flood safety pattern.

## 2. Background of Research

The rain and flood safety pattern is a part of the water security pattern research while the water security pattern is a part of the ecological security pattern research. The ecological security pattern is a concept proposed by Kongjian Yu based on the theory of landscape ecology in 1996 [9]. It refers to the spatial pattern composed of parts, points, and spatial relationships that play a key role in maintaining ecological security [10], which is a spatial pattern plan based on the interaction analysis ecological process [11]. In 2005, Yu and other scholars took the water security pattern as a study from the concept of ecological security pattern for the first time in the book “The “Anti-Planning” Approach” [12]. The water security pattern belongs to a section of the ecological security pattern research which focuses on the spatial pattern set with water ecological elements. It is based on the research and analysis of hydrological effects within a specific time and space range, which when guided by water security issues, and coupled research on water resources, water ecology, water environment, and water disasters [13], through a comprehensive multi-disciplinary and multi-perspective analysis of the key spatial unit elements to protect water safety, the effective spatial configuration of the key elements is realized [14]. In the book “Regional Ecological Security Pattern: Beijing Case” published in 2009, the spatial analysis module and hydrological module of ArcGIS were used for the first time to analyze and simulate the process of flood and surface runoff, thereby constructing the rain and flood safety [10]. This can be described in other words as the storm flood security pattern based on the mutual feedback of ecological and urban system, through the construction of the security pattern, to achieve effective regulation of the storm flood process [15]. The current global research on urban rainwater regulation and storage capacity is more inclined to think about rainwater issues from a systematic and comprehensive perspective and establish a sound technical framework [16]. Some scholars believe that the function of rainwater regulation and storage cannot be realized by only relying on the technical transformation and improvement of a single point. It must rely on all the green space systems in a region for overall coordination and common development, so as to enhance the rainwater management function of the green space [17]. Therefore, more and more scholars use the method of urban storm flood model construction to study the storm flood regulation and storage capacity of urban green space systems [18].

Emphasizing the management of flood risk in urban water systems, and then improving the resilience of urban water systems is considered to be the key to improving urban ecological resilience [19]. As an important component of the water security pattern, the rain and flood security pattern emphasizes that the flood disaster problem cannot be solved simply by flood control dams or simply by water transfer, but a comprehensive, holistic, and multi-objective solution to the problem should be established. The plan, by identifying the key locations and spatial connections in the process of flood control, establishes a security pattern for stormwater management [20]. One can determine the spatial layout of the flooded area in the storm flood process by judging and identifying the key location of the storm flood confluence and then constructing the rain and flood safety pattern according to the storm flood spatial distribution characteristics to achieve effective control of the storm flood process [21].Considering the connotation of the water security pattern and the ecological security pattern, the storm flood security pattern is the process of storm flood occurrence and its feedback on urban and rural security. It is divided into two parts: flood security pattern and waterlogging security pattern, involving the design of storm flood corridors: road, rain flood inundation safety pattern, rain flood pollution safety pattern, and other dimensions [22]. Under the current background of land and space planning, the core connotation of stormwater safety pattern construction is “Policy Integration”. Here, “coordination” means that different planning elements are not only coordinated and conflict-free in spatial layout, but more importantly, they can lead to the same goal and result [23], unify the goal of stormwater safety with other planning goals, and combine rigid control and flexible guidance, so that the elements of stormwater safety and other planning elements form a result-oriented linkage mode.

The current research on the storage capacity of rainwater and flood storage in green areas mainly focuses on the total volume. Few studies have evaluated the fine-scale changes in surface runoff reduction of green areas. In addition, most academic circles focus on the ability of a certain factor to adjust and store rainwater and flood in green areas. There is a lack of research on the influence mechanism of these internal factors on the rain and flood regulation and storage capacity of green land. The purpose of this research is to construct and analyze the rain and flood safety pattern in the central city of Harbin and propose planning recommendations based on the evaluation results to enhance the internal dynamic balance against storms and floods and put forward rain and flood regulation measures in a multi-scale environment. It also plays an extremely important role in preventing and draining waterlogging from disasters and improving the coordination of water ecosystem service functions with social and economic development. It provides methodological reference and reference for the construction of rain and flood safety patterns in future urban and rural development and planning.

## 3. Study Area and Data Sources

### 3.1. Study Area

**Figure 1:**
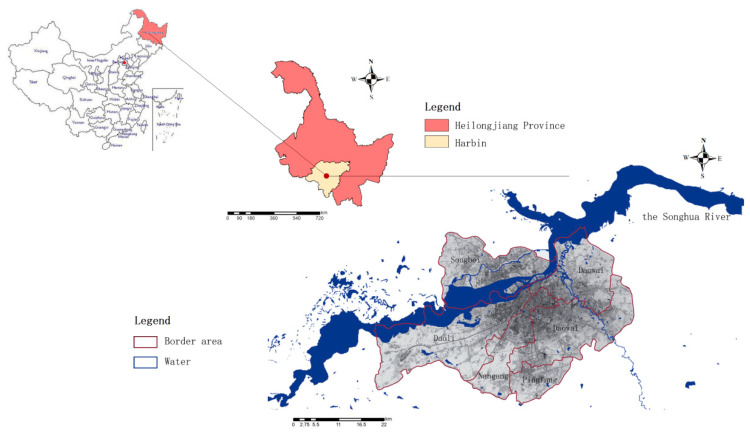
Schematic of study area.

Harbin is located in the northeastern part of China’s Northeast Plain, between 125°42′~130°10′ east longitude and 44°04′~46°40′ north latitude, which in the southern part of Heilongjiang Province and the central area of Northeast Asia. It is the first important hub for the Eurasian Land Bridge and air corridor. The climate of Harbin is a mid-temperate continental monsoon climate, with long winters and short summers. The annual average precipitation is 569.1 mm. The precipitation is mainly concentrated in June to September, and summer accounts for 60% of the annual precipitation [24]. The main distribution roads in the urban area of Harbin are on the third-level terraces formed by the Songhua River: the first-level terraces are between 132 and 140 m above sea level, mainly including Daoli District and Daowai District, with flat ground; the second-level terraces are 145–175 m above sea level. It gradually transitions from the first terrace without obvious boundaries, mainly including parts of Nangang District and Xiangfang District. It has a large area, long-term water erosion, slight undulation, deep soil layer, and fertile soil [25]. It is an important agricultural area in Harbin. The third-level terrace is 180–200 m above sea level, mainly distributed in the southern part of Huangshanzuizi and Hefang District, and then to the southeast, it gradually transitions to the Zhangguangcai Ridge, which is a hilly area [26]. In this study, six major urban areas, including Xiangfang District, Songbei District, Nangang District, Daoli District, Daowai District, and Pingfang District, which are densely populated in the central urban area of Harbin, were selected for analysis and research (Figure 1).

### 3.2. Data Sources

There are many types of data needed in this research. The core data sources are Landsat-8 OLI_TIRS satellite digital products, ASTER GDEM 30 M resolution digital elevation data, meteorological data, land use data, and administrative division data. All data sources are shown in Table 1.

**Table 1:** Core data sources list.

Data	Time	Format	Source	Resolution
Landsat satellite data	2020	Raster	Geospatial Data Cloud [27]	30 m × 30 m
Digital Elevation Model data	2018	Raster	Geospatial Data Cloud [27]	30 m × 30 m
Meteorological data	1957–2021	Text	Resource and Environment Science and Data Center [28]	_
Landuse data	2020	Raster	Resource and Environment Science and Data Center [29]	30 m × 30 m
Administrative division data	2015	Shapefile	Amap [30]	_

## 4. Methodology

**Figure 2:**
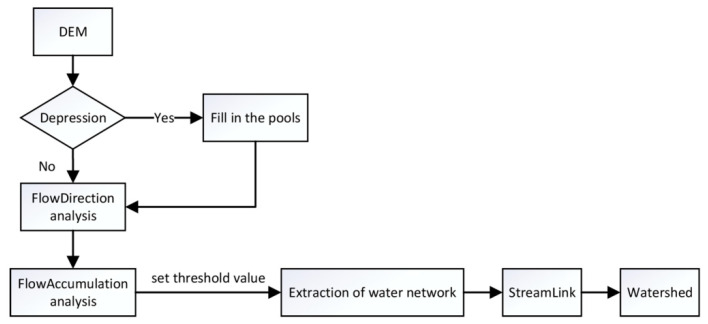
Process of hydrological spatial analysis.

The river system has a certain capacity for regulation and storage. The point source reservoir and the river system form a spatial corridor. Based on the GIS spatial analysis, a reasonable buffer design is carried out in combination with the city’s spatial control planning and flood control standards. The safety level evaluation is made according to the buffer position and the hazard level [31]. This study uses landscape ecology theory and relevant knowledge of ecological security pattern, takes the central urban area of Harbin as the research object, uses remote sensing, ArcGIS, and other spatial analysis techniques to quantitatively study rain and flood safety, and finally focuses on flood control and drainage and ecological protection and development. The planning strategies will be proposed from the perspective of building a scientific and reasonable rain and flood safety pattern. This method avoids complex quantitative relationships and can be combined with the flexibility of GIS to achieve spatial visualization of results. Important parameters such as remote sensing images and hydrological data can be directly obtained from public data on the Internet, which is free from the constraints of research data. This paper uses GIS technology to import the DEM digital elevation model into ArcGIS 10.8 and conduct simulation runoff analysis and a series of hydrological analysis to determine the range of depressions with potential rainwater storage functions, as well as the catchment points and outlets of basins, which are of key significance. Finally, the river network and watershed evaluation units in the central urban area of Harbin were extracted. Figure 2 is a flow chart of hydrological analysis based on GIS.

It can be seen from Figure 2 that the preprocessing in the hydrological spatial analysis process is slope analysis and depression filling. After the preprocessing is completed, DEM data can be used to generate watersheds. The water flow and other substances are sent from a public water outlet to form a concentrated drainage area. The grids in the same watershed can be linked by the direction of the water flow.

### 4.1. Analysis of Related Concepts

#### 4.1.1. Rain Flood Corridor

As a part of the stormwater security pattern, the stormwater corridor is a circulation channel where the stormwater flows and converges. The concept of “corridor” is based on the research of ecological security pattern. Constructing an ecological security pattern by identifying and analyzing the “source-corridor” is a common method for constructing an ecological security pattern. It is a research method with relatively simple data requirements, high computational efficiency, and visualization of results [32]. Among them, the ecological corridor refers to the belt-shaped area that has an important connection effect on the flow of material, information, and energy in the ecological network system, especially the circulation channel that has a key role in the migration of animals and plants [33]. In the process of rainfall, surface runoff converges and stays to produce urban waterlogging. The stormwater runoff pathway is an important area in the process of generating rain and flood safety patterns and stormwater convergence. Therefore, stormwater corridors are an important part of the construction of rain and flood safety patterns.

#### 4.1.2. Rain Flooded

In the process of rainfall, a large amount of rainwater is produced. This rainwater quickly converges in time and space to form waterlogged water on the surface. Rain flooding refers to the surface area covered by waterlogged water from the occurrence to the disappearance of the waterlogging. Characteristic attributes include duration, connectivity, frequency, depth, and range [34]. Among them, the rain flood inundation range refers to the area of the ground surface that was inundated when the city flooding disaster occurs. The rain flood inundation depth refers to the height from the surface of the underlying land surface to the surface of the stagnant water at a certain location, that is, the depth of the stagnant water [35]. The loss degree of rain flood disaster is closely related to the rain flood inundation status, including factors such as rain flood inundation depth, inundation range, inundation duration, and flow velocity during flooding [36]. Under normal circumstances, the wider the rain flood inundation area and the deeper the rain flood inundation depth, the greater the loss caused by the rain flood disaster. Therefore, rain flood inundation is an important part of the storm flood security pattern.

### 4.2. Method

#### 4.2.1. SCS Model

On the macro-scale, the current research using more quantitative analysis methods is based on factor weight evaluation and analysis, based on historical inundation data to construct the sponge city pattern, and relatively few studies on the use of hydrological models to simulate macro-scale waterlogging problems; most of the studies are on floods. From the perspective of urban planning, the SCS (Soil Conservation Service) model has great advantages in the macro-scale quantitative research and analysis of planning based on hydrological model simulation. The SCS model is a rainfall runoff model developed by the Water and Soil Conservation Service of the United States Department of Agriculture in 1954. The model has a simple structure and few parameters [37] and mainly studies the effects of soil type, land use, soil moisture, and other factors on rainwater runoff [38]. Due to different land use types, underlying surface soil types, and previous soil moisture levels, the impact of surface infiltration is different. The model parameters take into account these effects on rainfall and surface runoff, and it is a better method for calculating runoff in small catchments [39]. Using the joint analysis of the SCS hydrological model and the GIS spatial model, the spatial location of the urban rainstorm inundation area is calculated. With reference to variable factors such as rainstorm rainfall and surface runoff coefficient, the volume generated by the confluence per unit area can be calculated. The basic principle of the model is based on the water balance equation and the assumption of the relationship between the assumption of equal proportions and the initial loss value (the maximum potential detention at the time). The main calculation equations are as follows:

(1) $$P=I_{a\;}+F+Q$$

(2) $$\frac{Q}{P-I_{a}}=\frac{F}{S}$$

(3) $$I_{a}=\lambda \cdot S$$

P ≥ 0.2S:

(4) $$Q=\frac{{\left(P-0.2S\right)}^{2\;}}{P+0.8S}$$

P < 0.2S:

(5) Q=0

where P is the total rainfall (mm); Q is the direct runoff (mm); is the initial loss (mm), including interception, surface water storage, etc.; F is the cumulative infiltration (mm) not included; S It is the maximum detention possible (mm) at that time.

From Equations (4) and (5), it can be seen that the runoff depends on the rainfall P and the potential infiltration amount S before the rainfall in the field, and the potential infiltration amount S is also related to the soil quality in the study area.

Land and land use are related to the soil moisture before rainfall and other characteristics, and the S value has a large, varied range, which is not convenient to take the value. For this reason, the SCS model uses an empirical dimensionless parameter CN (curve number) that can comprehensively reflect the regional characteristics before rainfall to derive the S value. The calculation formula is:

(6) $$S=\frac{25400}{\mathrm{CN}}-254$$

CN is a dimensionless curve parameter describing the relationship between rainfall and runoff. It reflects the runoff generation capacity of the underlying surface of the basin. The influencing factors include the degree of soil moisture in the previous period, soil type, and land use type.

#### 4.2.2. Model Parameter Adjustment and Verification

According to the Nash–Suttcliffe coefficient, detecting the degree of agreement between the simulated value and the monitored value and using the linear correlation degree of the simulated curve and the monitored curve to determine the simulation accuracy is one of the common methods for model parameter adjustment and verification. The Nash–Suttcliffe coefficient is a non-dimensional parameter. The value range is generally between 0 and 1. The closer the value is to 1, the more credible the result is, and the closer the value is to 0, the less reliable the result is [40]. The Nash–Suttcliffe coefficient is used to quantitatively characterize the simulated and monitored values of CN parameters in the SCS model and adjust the model’s fitting degree to the entire meridian to make the parameter values more accurate. The principle formula is:

(7) $$E_{ns}=1-\frac{{\sum^{\text{​}}}_{i=1}^{n}{\left(Q_{o}-Q_{p}\right)}^{2}}{{\sum^{\text{​}}}_{i=1}^{n}{\left(Q_{o}-Q_{\mathrm{ave}}\right)}^{2}}$$

Among them is the Nash–Suttcliffe coefficient, which is the actual measured value at the *i*-moment, is the simulated value at the *i*-moment, and n is the total number of periods, which represents the measured average value. Select the measured rainfall value, use the initially determined CN parameter value to simulate the runoff value according to the SCS model, compare the measured rainfall value with the simulated values, adjust the model parameters several times, and finally obtain the CN value when the Nash–Suttcliffe coefficient is close to 0.9.

## 5. Results and Discussion

### 5.1. Data Preprocessing

**Figure 3:**
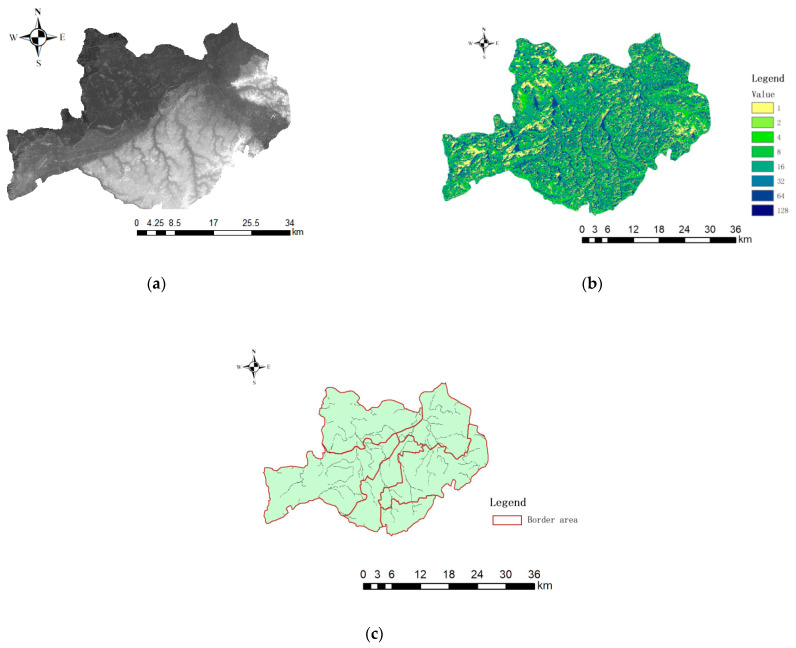
(**a**) DEM map without depression. (**b**) Flow direction extraction diagram. (**c**) River network extraction map.

Due to the existence of special topography in the inner area, there are often some unreasonable depressions on the surface of DEM data, called depressions. If these depressions are not treated, the wrong water flow direction will occur in the hydrological analysis and the analysis will be generated error. Therefore, before analysis, the original DEM data should be calculated for depressions, the most suitable threshold for the study area should be determined according to the calculation results, and depressions should be filled to obtain DEM without depressions in the study area (Figure 3a).

In ArcGIS, the water flow direction uses the D8 single flow direction algorithm. This method judges the water flow direction based on the high and low values of each grid. It assumes that the water flow in the grid can only flow out from one direction and enter another grid through the calculation center The maximum distance weight drop between the grid and the neighborhood grid is assigned to different grids. The value of the maximum distance weight drop represents the distance between the grids, and the distance determines the direction of the water flow. This paper uses the hydrological analysis module of ArcGIS software to extract the direction of water flow in the study area (Figure 3b).

According to the basic principle of the confluence accumulation, when the confluence accumulation reaches a certain value, there will be an obvious water flow. By setting the threshold, the potential path of the water flow under the corresponding confluence accumulation can be obtained. When these paths are large enough, it constitutes a river network. In the process of river network extraction, the threshold value that can produce the smallest difference should be selected to reduce the error. Use ArcGIS software “Spatial Analyst”-”Map Algebra”-”Raster Calculator” tool to complete the river network extraction operation and convert it into a vector river net in order to obtaining the river network map in the study area (Figure 3c).

### 5.2. Dividing Watersheds

**Figure 4:**
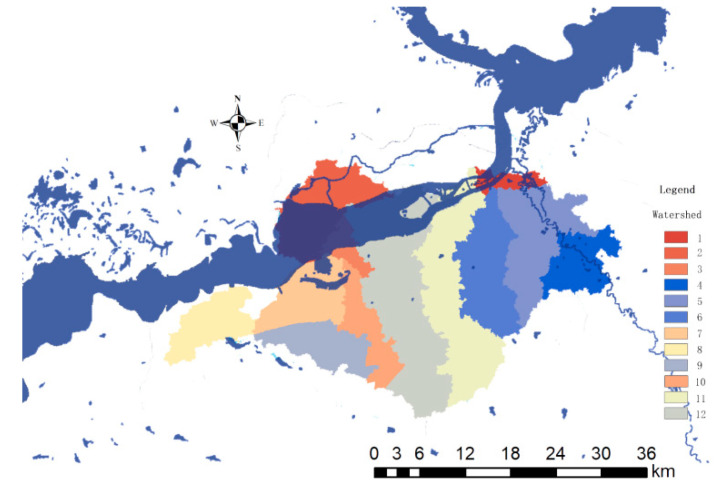
Extraction map of watershed evaluation unit.

Firstly, determine the largest watershed in the study area, use the Watershed function to search for the watershed boundary on the water flow direction grid, and organize and modify the extracted data within the watershed area, filter out the sub-watersheds within the study area, and get the study distribution map of district sub-watersheds (Figure 4). Through the extraction of small watersheds, the study is divided into 12 sub-basin evaluation units for evaluation.

### 5.3. Runoff Generation and Runoff Calculation Based on SCS-CN Model

#### 5.3.1. Model Parameter Determination

The CN value is a comprehensive parameter that can characterize the characteristics of land use type and hydrological soil. It is combined by three factors such as the degree of soil moisture in the early stage, soil type, and land use method. It is often obtained according to the CN value lookup table of the National Engineering Manual of the United States [41]. Since the classification of land use types in the United States and China adopt different standards, and the climatic environment of the United States and China are also quite different, the names of urban land use and the CN value corresponding to the SCS model will change. This study is based on the land name of the planning map of the study area, combined with the CN value of the study area SCS model; refer to the reference value of the American engineering manual, according to different underlying surface types, combined with the research results of the adjacent similar areas in the relevant literature, select the initial value, and then combine the actual measurement. Values are adjusted and verified, the parameters are calibrated, and the CN parameter values are determined as shown in the table below. The China Meteorological Administration stipulates that the rainfall within 24 h is called daily rainfall. Any day rainfall below 10 mm is called light rain, 10.0–24.9 mm is moderate rain, 25.0–49.9 mm is heavy rain, and rainstorm is 50.0–99.9 mm. The heavy rainstorm is 100.0–250.0 mm, and the rain exceeding 250.0 mm is called the extra heavy rainstorm. This paper selects 50 mm precipitation when heavy rain occurs as the data for research, and the calculated data is shown in Table 2.

#### 5.3.2. Rainwater Runoff Calculation

Based on the statistics of land use types and direct runoff, and according to the SCS-CN model, the total runoff generated by various types of land is calculated, as shown in Table 3. It can be seen from Table 3 that the total amount of runoff generated by the No. 12 and No. 11 basins in the study area is 25.47% and 17.3% of the total respectively.

#### 5.3.3. Rainwater Runoff Simulation

**Figure 5:**
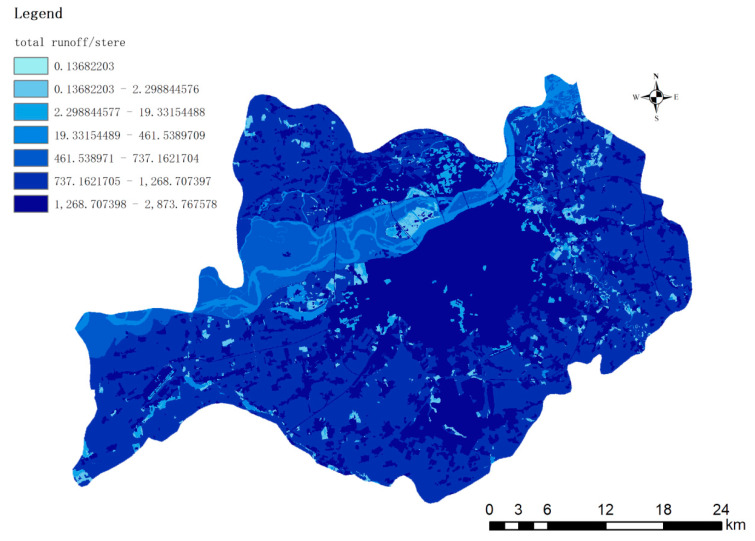
Total runoff space distribution.

Based on the spatial analysis function of ArcGIS, the data in Table 3 is imported into GIS to simulate the spatial distribution of runoff, and the results are shown in Figure 5.

Combining the direct runoff statistics in Figure 5 and Table 3, it can be seen that after a single rainfall of 50 mm of precipitation, the regional soil and climate conditions of Harbin City, combined with the empirical parameters of the different land use of the SCS-CN model, it can be seen intuitively that the artificial land Areas No. 11 and No. 12 with the largest surface area have the largest runoff. However, the percentages of man-made surfaces in these two areas are not the highest. The percentages of man-made surfaces in No. 6 and No. 11 are respectively 73.5% and 64.8%, both of which are located in Xiangfang District, where has many factories. The top two areas with traffic proportions are the No. 12 and No. 11 basins. The No. 12 basin is located in Xinghuo Industrial Park, Hashuang Road, near Hejiagou. Comparing the No. 12 basin with the highest and lowest proportion of the flow with the No. 3 basin, the average runoff coefficient of the No. 3 basin is 39.94, which is higher than the 36.45 of the No. 12 basin. The coordinates of the No. 3 basin are converted using ArcGIS and it is found that the basin is located Near Diding Road in Daoli District, bordering the Songhua River; the percentage of water in the basin is the highest at 57.7%, which seriously affects the value of the runoff coefficient.

#### 5.3.4. Rain Flood Inundation Risk Rating

**Figure 6:**
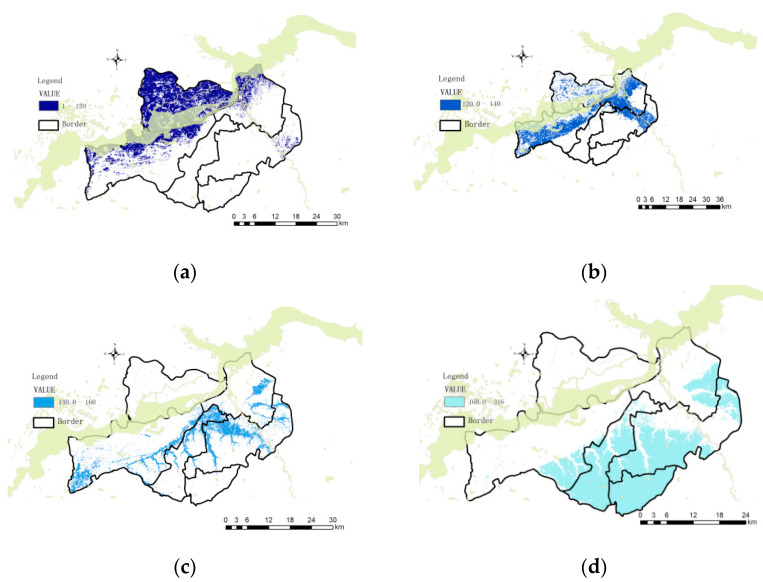
(**a**) High-risk areas. (**b**) Higher risk areas. (**c**) Medium-risk areas. (**d**) Low-risk areas.

Combining the historical data of rain and flood in Harbin City, the rain and flood disaster risk area in the study area is delineated, and the result is shown in Figure 6.

As shown in the figure, according to the evaluation results, Songbei District has the lowest rain and flood safety factor in the study area, and the bungalow area has the highest safety index. The area has the highest rainwater safety level. In addition to the geographical advantages, it also has the advantages of the type and distribution of land use. As for the water permeability and coefficient of urban land use, further research is needed. Among the watersheds divided, the watershed 2 in Songbei District has the highest risk. Except for the low-lying terrain, the water in this area accounts for 41.1%, which increases the average runoff in the area and increases the risk.

### 5.4. Stormwater Safety Optimization Strategy Based on the Concept of Resilient Urban and Rural Areas

The rain and flood safety pattern can be used as an inaccessible rigid limit boundary for urban expansion, limiting the disorderly expansion of the city, and at the same time, from the perspective of space utilization, guiding urban land use and development and ensuring personal safety [42]. Spatially designing a safety protection area for high-risk areas and making planting coverage tailored to local conditions in the corresponding areas ensures the ecological balance in the low-safety pattern and the safety of residents’ activities. Strengthening soil penetration and filtration through plant planting promotes connectivity between water bodies [43]. From the perspective of urban planning, in the protection and control of low-security patterns, it is necessary to ensure that the land in the area is non-construction land and has sufficient space for flood discharge. In the specific construction of a city, it is necessary to refer to the rain and flood safety pattern, make guiding planning and design on the urban spatial scale, propose appropriate engineering optimization measures according to the rain and flood safety pattern of different security levels, and use natural water network output, regulation, and storage. Purification function can reduce the construction cost of artificial drainage pipe network facilities, reduce the burden of urban drainage, and improve the ability of the original urban drainage system to resist rain and flood [44]. In the process of planning, design, layout and development, and construction, refer to the evaluation results of rain and flood safety pattern, protect the original storm flood corridors, strengthen the connection between storm flood control patches, ensure the connectivity of storm flood corridors, and construct natural storm flood regulation. The storage system will increase the city’s ability to withstand the risk of heavy rain, reduce the damage caused by urban waterlogging, and achieve the goal of sponge city construction.

## 6. Conclusions

Urban storm flood disaster prevention and control is a complex systematic project, involving various environmental levels from the macro to the micro level, as well as various influencing factors such as urban economy and society. In operation, it is necessary to comprehensively use system thinking for thinking. In this study, the SCS-CN model was used to more accurately calculate the rainwater runoff distribution of various urban land after a single rainfall of 50 mm in the Songbei District, Daoli District, Nangang District, Xiangfang District, Pingfang District, and Daowai District of Harbin. Data: based on the combination of GIS spatial simulation technology and the SCS-CN model, the distribution of rainwater runoff in the urban space is shown, and the plots prone to water accumulation are more intuitively displayed. Spatial simulation of the study area, according to the topography, intuitively reflects the land prone According to the spatial simulation of the study area based on the topography can intuitively reflect the flood-prone areas, which is helpful to provide targeted reference opinions for the construction of sponge cities.

Under the guidance of the planning framework summarized in the previous article, following the principles and models of regulating and controlling cold water basin water systems from the perspective of storm and flood management, the specific ways for watershed water systems to achieve storm and flood management are proposed: (1) The operation of dividing the study area into small watershed, constructing a more accurate security pattern for corresponding analysis, so as to ensure the stormwater security of the city; (2) After exploring the interaction among different regions, the storm flood problem is allocated to each region, and different storm flood management tasks are proposed according to the regional characteristics, so as to achieve the overall goal of safe stormwater management.

Combining ArcGIS spatial analysis tools with hydrological models for simulation research, constructing a rain and flood safety pattern from the perspective of flood control and drainage, predicting the key points of storm flood management, and providing early warning for future storm flood safety management.

## Figures and Tables

**Table 2:** SCS model parameter table.

No.	Type of Underlying Surface	CN Value	Potential Infiltration (S)/mm	Direct Surface Runoff (Q)/mm
1	Farmland	80	63.5	13.80
2	Forest	56	199.57	0.49
3	Grass	66	130.85	3.67
4	Water	100	0	50
5	Artificial surface	98	5.18	44.28
6	Bare land	86	41.35	20.96

**Table 3:** Statistics of land use and the total runoff of the study area.

No.	Number of Patches	Area/km^2^	Percent of Total Area/%	Average Runoff (Q/mm)	Total Runoff/m^3^	Runoff Percent of Every Land Use Type/%
1	251	19.37	1.78%	30.19	584,780	1.77%
2	419	106.44	9.79%	36.07	3,839,290	11.6%
3	122	22.20	2.04%	39.94	886,668	2.68%
4	412	60.54	5.57%	20.24	1,225,330	3.7%
5	1278	116.26	10.7%	24.51	2,849,533	8.61%
6	342	104.52	9.62%	35.93	3,755,404	11.34%
7	671	76.82	7.07%	23.38	1,796,052	5.43%
8	337	67.41	6.20%	19.68	1,326,629	4.01%
9	162	65.59	6.04%	21.12	1,385,260	4.18%
10	202	54.45	5.01%	23.82	1,296,999	3.92%
11	336	161.85	14.89%	35.37	5,724,634	17.3%
12	971	231.26	21.29%	36.45	8,429,427	25.47%
Total	5503	1086.71	100%	346.7	33,100,006	100%
